# Rice callus suspension culture inhibits growth of cell lines of multiple cancer types and induces apoptosis in lung cancer cell line

**DOI:** 10.1186/s12906-016-1423-3

**Published:** 2016-11-02

**Authors:** Nafeesa Rahman, Surendar Reddy Dhadi, Aparna Deshpande, Wusirika Ramakrishna

**Affiliations:** 1 Department of Biological Sciences, Michigan Technological University, Houghton, MI USA; 2 Centre for Biochemistry and Microbial Sciences, Central University of Punjab, Bathinda, Punjab India

**Keywords:** Cancer cell line, Rice callus culture, Cell viability, Apoptosis, Lactate dehydrogenase

## Abstract

**Background:**

Cancer is one of the leading cause of mortality. Even though efficient drugs are being produced to treat cancer, conventional medicines are costly and have adverse effects. As a result, alternative treatments are being tried due to their low cost and little or no adverse effects. Our previous study identified one such alternative in rice callus suspension culture (RCSC) which was more efficient than Taxol® and Etoposide, in reducing the viability of human colon and renal cancer cells in culture with minimal or no effect on a normal cell line.

**Methods:**

In this study, we tested the effect of RCSC by studying the dynamics of lactate dehydrogenase (LDH) in lung cancer cell lines (NCI-H460 and A549), breast cancer cell lines (MDA-MB-231 and MCF-7) and colorectal cancer cell lines (SW620 and Caco-2) as well as their normal-prototypes. Complementary analysis for evaluating membrane integrity was performed by estimating LDH release in non-lysed cells and cell viability with WST-1 assay. Fluorescence microscopy with stains targeting nucleus and cell membrane as well as caspase 3/7 and Annexin V assays were performed. Real-time quantitative RT-PCR was performed to evaluate expression of 92 genes associated with molecular mechanisms of cancer in RCSC treated ling cancer cell line, NCI-H460 and its normal prototype, MRC-5. High performance liquid chromatography (HPLC) was used to collect RCSC fractions, which were evaluated on NCI-H460 for their anti-cancer activity.

**Results:**

Lower dilutions of RCSC showed maximum reduction in total LDH indicating reduced viability in majority of the cancer cell lines tested with minimal or no effect on normal cell lines compared to the control. Complementary analysis based on LDH release in non-lysed cells and WST-1 assay mostly supported total LDH results. RCSC showed the best effect on the lung non-small carcinoma cell line, NCI-H460. Fluorescence microscopy analyses suggested apoptosis as the most likely event in NCI-H460 treated with RCSC. Gene expression analysis identified significant upregulation of cJUN, NF-κB2 and ITGA2B in NCI-H460 which resulted most likely in the arrest of cell cycle progression and induction of apoptotic process. Further, HPLC-derived RCSC fractions were less effective in reducing cell viability than whole RCSC suggesting that a holistic approach of using RCSC is a better approach in inhibiting cancer cell proliferation.

**Conclusions:**

RCSC was found to be an effective anti-cancer agent on cell lines of multiple cancer types with the best effect on lung cancer cell lines. A possible mechanism for the anticancer activity of RCSC is through induction of apoptosis as observed in the lung cancer cell line, NCI-H460.

**Electronic supplementary material:**

The online version of this article (doi:10.1186/s12906-016-1423-3) contains supplementary material, which is available to authorized users.

## Background

The incidence of cancer has been reported to increase with age. Estimated cases reported for lung, breast, and colorectal cancers are 1.82, 1.67 and 1.36 million, respectively [1]. Among these, lung cancer caused the most number of deaths at 1.6 million. Cancer incidence is estimated to increase every year reaching 26 million new cases and 17 million deaths per year by 2030 [2]. A diagnostic feature of cancer is uncontrolled cell proliferation, which is normally maintained by mutational events that regulate various signaling and metabolic pathways. Transformation of a normal cell to tumor cell requires continuous growth signals, resistance to apoptosis, the ability of angiogenesis and finally tissue invasive and migratory capability. Eradication of cancer seems inevitable since the core cause of its pathogenesis is the rapidly changing cell machinery. The reason behind these mutations is different from one cell to another and one patient to another. With the ever-increasing incidence of cancer, the need for better and efficient drugs is a target of most cancer researchers. Since cancer cells tend to use their normal signaling and metabolic machinery to proliferate and progress, it is difficult to specifically target cancer cells. The adverse effects due to the targeting of normal cells surrounding cancer cells render these drugs ineffective. Identifying compounds specifically targeting cancer cells have therefore been the main focus of researchers over the last two decades.

Plants have been a direct/indirect source of medicine in the past and their medicinal properties continue to be investigated and exploited all over the world [3–7]. The World Health Organization (WHO) has estimated that about 80 % of the human population rely on herbal drugs than on conventional synthetic drugs [8]. The bioactive components of plants have been documented as potential anti-cancer agents. This is supported by the fact that about 60 % of the prescribed anti-cancer drugs are either synthetic or semi-synthetic derivatives of plant metabolites. These include paclitaxel derived from Pacific yew, vinca alkaloids like vincristine and vinblastine from Madagascar periwinkle, and etoposide from *Podophyllum* species. Despite advances in the development of synthetic oncogenic drugs, researchers are continually trying to identify plant-derived metabolites that effectively target cancer cells, have high efficacy with minimal/no adverse effects, non-toxic to healthy tissues and systems, readily available, and affordable. Indeed, some plant metabolites have been reported in targeting cancerous cells with minimal or no effect on normal cells. For example, extracts of *Kalanchoe tubiflora* showed cell cycle arrest and senescence-inducing effect against various cancer cell lines [9] and *A. monosperma* [10] showed anticancer effects against various hematological and solid tumor cell lines by inducing apoptosis. They comparatively showed minimal or no effects on normal healthy cell cultures. These effects are therefore considered better compared to the toxicity of drugs like taxanes and vinca alkaloids have on normal cell lines.

Plant callus has been a new target of research because they harbor metabolites with significant health benefits. Callus is a mass of somatic undifferentiated totipotent cells. Callus extracts were found to perform better than extracts from plant parts against various diseases. For example, *Aegele marmelos* whole callus extract lowered blood sugar more efficiently than the leaf extract [11]. The overall synergistic effects of various compounds in the callus extract could be the possible reason for this observation. Rice callus suspension culture (RCSC) was shown to harbor anticancer activity with 95 % and 87 % reduction in cell viability of a renal cancer cell line (RXF-393) and a colon cancer cell line (SW620), respectively, in a dose and time-dependent manner, with minimal inhibition of normal lung fibroblast cell line (MRC-5) [12]. However, only once cell line for each of the two cancer types was used and the corresponding normal cell lines for renal and colon cancer were not evaluated in the above report.

The most common cancer types in the United States include lung, breast, and colorectal cancers (https://www.cancer.gov/about-cancer/understanding/statistics). In the current study, the anticancer activity of different dilutions of RCSC was tested on these three cancer types using two human cell lines for each cancer type and corresponding normal cell lines. The cancer type that showed the best response to RCSC treatment was characterized with reference to apoptotic markers, and the expression of specific cancer-related genes.

## Methods

### Rice callus suspension culture (RCSC) and plant medium (PM) preparation

Liquid plant medium (PM) was prepared first by dissolving Murashige and Skoog (MS) media (PhytoTechnology Lab) in deionized water supplemented with proline, casamino acids and sucrose and autoclaved for 20 min at 125 °C. After cooling, it was further supplemented with 2,4D (2,4 dichlorophenoxyacetic acid) and Chu’s N6 vitamins (PhytoTechnology Lab). RCSC was prepared as described earlier [12]. Briefly, rice callus was produced from seeds grown in sterile solid plant medium followed by their transfer to sterile liquid plant medium. The calli were allowed to grow and exude compounds in the liquid medium for three weeks. At the end of three weeks, UV absorbance between 240 nm and 300 nm was measured using Nanodrop. RCSC was then centrifuged at 10,000 rpm for 10 min at 4 °C to remove particulate matter and cell debris. The clear supernatant was filter-sterilized and aliquoted aseptically into 50 mL sterile culture tubes and stored at −20 °C for further use.

### Human cancer cell lines, culturing, passaging and seeding

The cell lines selected for this study were human lung cancer cell lines (NCI-H460 and A549), breast cancer cell lines (MCF7 and MDA-MB-231), and colorectal cancer cell lines (SW620 and Caco-2). Normal prototypes used as control were lung fibroblast (MRC-5), human mammary epithelial cell line (HMEC) and normal colon mucosal cell line (NCM356). All cell lines, except NCM356, were obtained from ATCC (American Type for Culture Collection). NCM356 was licensed from INCELL Corporation (San Antonio, TX). Cell culturing media was obtained from ATCC or INCELL.

The cell lines were cultured in their respective media, namely, Dulbecco’s minimal essential medium (DMEM) for MDA-MB 231, Eagle’s minimum essential medium (EMEM) for MCF-7, Caco-2, and MRC-5), Roswell Park Memorial Institute (RPMI-1640) for NCI-H460 and SW620, M3:BaseA culture media for NCM-356, F-12 K for A549, and mammary epithelial cell base medium for HMEC. Media was supplemented with Fetal Bovine Serum (FBS) to a final concentration of 10 % except for Caco-2 where it was 20 % as per manufacturers’ specification. Penicillin, streptomycin and amphotericin solution (ATCC) was added to the media diluted to 1000-fold (final). The cells were stored in liquid nitrogen vapor phase until further use. For cell culturing, corresponding vials were thawed by immersing them in a water bath at 37 °C for no more than 10 s. The cells were then immediately transferred to 100 × 20 mm sterile culture dish containing 10 mL of appropriate culture medium in the sterile cell culture hood. The plate(s) was then incubated at 37 °C at 5 % CO_2_ and 95 % relative humidity. Once the cells reached 80–90 % confluence, they were trypsinized with trypsin-EDTA (Hyclone) according to the manufacturer’s cell culture protocol. Briefly, first used cell culture medium was discarded and washed with 2 mL of trypsin-EDTA to remove any traces of medium. Then 3 mL of trypsin-EDTA was added and left for 5–10 min until the cell monolayer detached from the culture plate. Then trypsin was diluted with 7 mL of respective cell media. After trypsinization the cells were passaged, seeded or frozen depending on the experimental requirements.

### Cell counting and seeding protocol

Cells were counted in a hemocytometer using trypan blue stain. First, the cells were diluted in 1:20 ratio with cell culture medium. They were further diluted with trypan blue by adding 10 μL of stain to 50 μL of cells and counted in a hemocytometer. Number of cells per mL was calculated by using the formula: No. of cells/mL = (x) * (20) * (1.2) * 10^4^ where, x = average no. of cells in 4 squares, 20 = first dilution factor with cells and medium, 1.2 = second dilution factor with trypan blue. After each cell count, the volume of cells and medium needed for each experiment were taken and cells were seeded in each well of the 96 well flat bottom dark plate and incubated in a humidified 5 % CO_2_ incubator at 37 °C for 24 h before further experimental procedure.

### Cell freezing protocol

For freezing, the cell monolayer were first detached using trypsin-EDTA as mentioned above and transferred into 15 ml or 50 ml conical tube and centrifuged at 1100 rpm except for NCM-356 cells, which were centrifuged at 2000 rpm for 10 min. The cell pellet was suspended in freezing medium (10 % DMSO). Cells were stored first in −20 °C for 24 h followed by −80 °C for the next 24 h before storing it in liquid nitrogen.

### Treatment of cell lines with RCSC and PM

After the respective cell lines reached the desired confluence, they were seeded in a 96 well plate with cell densities of 10,000 cells/mL and 25,000 cells/mL in 100 μL volume. After 24 h of incubation, cells were treated in triplicate with different dilutions of filter sterilized RCSC or plant medium (PM). Five 2-fold serial dilutions (1:5, 1:10, 1:20, 1:40, and 1:80) of RCSC, corresponding to 0.25, 0.5, 1, 2 and 4 mg/mL of rice callus tissue were used to treat the cells for 24, 48, 72, and 96 h. In addition, three working concentrations of Taxol namely, 10^−5^ M, 10^−6^ M, 10^−7^ M, corresponding to 8.56, 0.856 and 0.0856 μg/mL were included as a positive control. Treatment with 0.05 % DMSO was included as a negative control. Stock solution (250 mM) of Taxol® was made in 100 % DMSO. At the end of the treatment period, cell proliferation assay, WST-1 and cytotoxicity assay (LDH assay) were performed. Cells were analyzed for morphological changes using an inverted light microscope at 5×, 10× and 40× magnifications.

### Cell viability assay

Cell viability assay was performed using pre-mixed WST-1 cell proliferation assay (Takara). WST-1 reagent contains a tetrazolium salt that is cleaved by mitochondrial reductase enzymes of a viable cell to produce yellow to orange colored formazan dye. Formazan is known to show an absorbance at 440 nm. The intensity of formazan dye depends on the number of viable cells or metabolically active cells. After each treatment period, 10 μL of WST-1 reagent was added to each well and plates were incubated at 37 °C at 5 % CO_2_ for 2 h. Absorbance was measured in a Multiskan plate reader (Thermo Scientific) at 440 nm. Percent decrease in cell viability was calculated from the absorbance of negative control or untreated cells as per the kit protocol. A two-sample *t*-test was performed using the function in Microsoft Excel to determine the statistical significance of the data at p < 0.05 and p < 0.01.

### Cytotoxicity assay

Cytotoxicity assay was performed using CytoTox 96 non-radioactive cytotoxicity assay kit (Promega). The total LDH (lysis) and the LDH released (non-lysis) from the cell due to changes in the cell membrane integrity were measured using this kit. The assay reagent contains tetrazolium salts that get reduced by the LDH of the cell to produce reddish formazan product, which is measured at 490 nm. The Total LDH content of the cell was measured by lysing the cell followed by the addition of a substrate buffer and quantification of red colored formazan produced by the reaction using Multiskan plate reader. The percentage of total LDH content and LDH released was measured as per the kit protocol. Statistical analysis was performed as described above for cell viability assay.

### Fluorescence microscopy

Cells were seeded into 96 well plates at a density of 25,000 cells/mL and allowed to acclimate for 24 h before treatment. The cells were then treated with selected 1:5 and 1:40 dilutions of RCSC and plant medium. Taxol and DMSO treatments were also included as positive and negative controls, respectively. Cells were subjected to treatment for 24 h and 72 h. At the end of the treatment period, the cells were stained with NucBlue live cell stain, Cell Mask plasma membrane stain (green), Alexa Flour 488 annexin V/dead cell apoptosis kit with Alexa® Fluor 488 annexin V and PI, and Caspase 3/7 stain (Life Technologies). NucBlue live cell stain is a Hoechst 33342 stain, which is highly cell permeable and binds DNA emitting fluorescence observed at 365 nm with an emission maximum of 460 nm. Cell mask PM stain is a lyphophilic stain that efficiently stains live membrane emitting fluorescence at 522 nm. NucBlue stain and Cell mask PM stain were used together to stain each seeded wells according to manufacturer’s protocol. AnnexinV binds to the externalized phosphatidyl serine (PS) staining it green and denotes the early stage of apoptosis. Propidium iodide (PI) stain is cell impermeant, which enters the cell only when the cell membrane is disrupted and stains the chromatin red indicating the loss of membrane integrity due to the cytotoxic effect of agents or due to ongoing necrosis process. Both Annexin and PI stain were used together according to protocol instructions. Caspase 3/7 stain is a cell-permeant DNA binding stain that emits green fluorescence when caspases 3 and 7 are activated.

Fluorescence emitted from NucBlue, AnnexinV and PI stains was observed at 365 nm with a Zeiss fluorescence microscope. Plasma membrane and caspase 3/7 activity detecting stains were observed at 470 nm. All the samples were observed at 10× and 20× magnifications. Image J software was used to quantify fluorescence by calculating corrected total cell fluorescence (CTCF) (http://imagej.nih.gov/ij/index.html).

### Scanning electron microscopic analysis

Preparation of SW620 for SEM was done using standard methods [13, 14] with some modifications. The colon cancer cells (5000 cells/mL) were grown on a sterile cover slip placed at the bottom of a polyvinyl coated petri dish. DMEM growth medium was supplemented with filter sterilized 1:5 RCSC dilution. After 96 h of treatment at 37 °C in 5 % CO_2_, the cover slips were removed, and the adherent cells were fixed using 1 % glutaraldehyde in 0.15 M phosphate buffer (pH 7.3) and incubated for 12 h. Cells were again washed 3–4 times in phosphate buffer and post fixed in 1 % osmium tetraoxide containing 0.15 % phosphate buffer (pH 7.3) for 1 h. Incubated cells were washed with buffer for 3–4 times followed by washes with sterile distilled water. The cells were then dehydrated in a series of graded ice cold alcohol (50 %, 80 %, 95 %, and 100 %), soaking the cells in each solution for 1 min. Once the dehydration was achieved, they were transferred to 100 % ethanol at room temperature and incubated for 20–30 min. The last step was repeated twice. The cells on cover slips were washed with acetone thrice and air dried at 28 °C. The cover slip with processed cells were then cut into small squares (5 × 5 mm) and glued onto polished aluminum stubs. The stubs were sputter coated with platinum nanoparticles 100A thick and observed under SEM.

### HPLC fractionation

RCSC and plant medium were fractionated using high performance liquid chromatography (HPLC). Fifty mL each of filter sterilized RCSC (corresponds to 20 mg callus/mL) and plant medium were lyophilized and dissolved in 2 mL of sterile deionized water to a final concentration of 500 mg/mL. Hundred microliters of each sample was injected in a C18 reverse phase column (Varian) and fractionation was performed using a photo diode array detector (Agilent Technologies). The total run time was 65 min. Compounds were separated in a binary gradient of 5 % acetic acid (solvent A) and 50 % acetonitrile (Solvent B). A total of eight fractions with retention time 2 min to 31.7 min were collected. Four fractions of RCSC that contained significant peaks on the chromatogram and were absent from the plant medium control were chosen for further analysis. The fractions collected were calculated as a percentage of the total area of peaks at 254 nm.

### Treatment with HPLC fractions and LDH assay

Cells were seeded in a 96 well plate at a density of 25,000 cells/mL and incubated for 24 h at 37 °C with 5 % CO_2_. After 24 h, cells were treated with three 4-fold serial dilutions (1:10, 1:40, and 1:160) of RCSC fractions as well as plant medium fractions. LDH assay measuring total LDH was carried out at four time points, namely, 24 h, 48 h, 72 h, and 96 h. The effects of selected HPLC fractions were compared with the same dilutions (1:10, 1:40; 1:160) of whole RCSC and plant medium at the four time points.

### Gene expression assay

For gene expression analysis, cells were first grown in their respective media until confluency, seeded in 6-well plates at a density of 25,000 cells/mL and treated with 1:5 and 1:40 dilutions of RCSC. The same dilutions for plant medium were used as negative control. Treatments were carried out for 24 h and 72 h. Total RNA was isolated from these samples using RNAeasy Plus Mini kit (Qiagen) according to manufacturer’s instructions. 25 ng of total RNA was used to make cDNA using SuperScript VILO Master Mix (Life Technologies). Quantitative RT-PCR was performed using GADPH and 18S TaqMan® probes and primers and TaqMan® Universal Master Mix and run on StepOnePlus Real-Time PCR System (Applied Biosystems). Three technical replicates and three biological replicates were used for each sample. The samples showing constituent expression in both GADPH and 18S were chosen for qPCR with TaqMan® Array 96 well fast plate (Applied Biosystems). These TaqMan® plates contain primers and TaqMan® probes for PCR amplification of 92 genes associated with molecular mechanisms of cancer and four endogenous control genes (GADPH, HPRT1 GUSB, and 18S rRNA). The plate was used to evaluate the relative expression changes of various cancer-related genes in cell lines treated with 1:5 and 1:40 dilution of RCSC for 24 h and 72 h. Fold changes in gene expression were calculated using the Data Assist software (Applied Biosystems).

## Results

RCSC affected cell proliferation and viability in a time- and dose-dependent manner. However, its effectiveness is strongly influenced by the type of cancer cell line used. In general, RCSC’s anti-proliferative effect was most pronounced after 72 or 96 h treatment. Treated cancer cells were found to be non-proliferative under an inverted light microscope, as there was no change in the cell density compared to the untreated control, which proliferated and grew in number crowding the culture plate at 96 h. Characteristic changes in the morphology of cancer cells were observed when compared to their corresponding negative and plant medium controls with a dramatic reduction in cell size and increased granulation as observed in NCI-H460 after 96 h treatment. The presence of secondary metabolites in RCSC are likely to be responsible for their anticancer effect.

### Different cancer types showed differential responses when treated with RCSC

#### RCSC treatment showed pronounced effect on metastatic colon cancer cell line as compared to non-metastatic colon cancer cell line

**Fig. 1:**
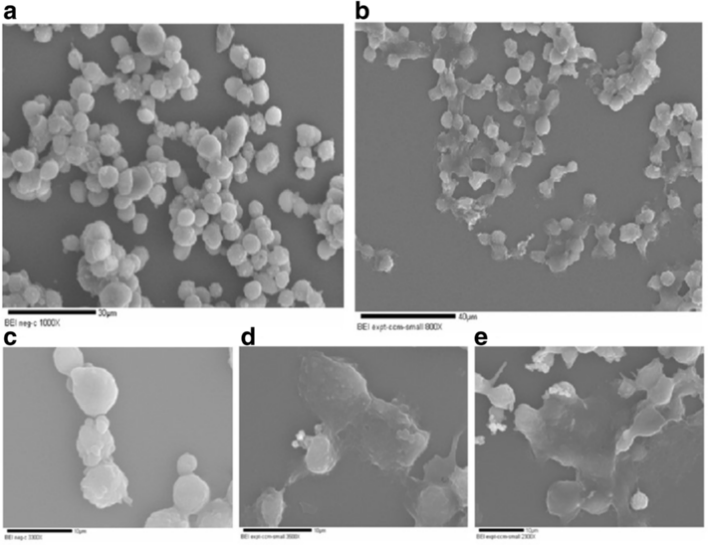
Scanning electron microscopic analysis of the colon cancer cell line (SW620) grown for 96 h with (**a**) no treatment (negative control) and (**b**) treated with 1:5 dilution of rice callus culture. Higher magnifications of the colon cancer cell line with (**c**) no treatment and (**d** and **e**) treated with rice callus culture

SW620 is derived from lymph node metastasis cells whereas Caco-2 is derived from colon epithelial cells (non-metastatic) [15]. Initial experiments with SW620 were carried out using two cell densities: 10,000 and 25,000 cells/mL. SW620 was more responsive to RCSC treatment at higher cell density. Lower dilutions (1:5 to 1:20) of RCSC significantly reduced total LDH in SW620 at a cell density of 25,000 cells/mL starting at 24 h (Additional file 1: Figure S1). The best effect was observed with 1:5 dilution (4 mg/mL) of RCSC, which reduced LDH by >90 % compared to the control after 96 h. The percentage reduction of LDH decreased with increase in dilutions of RCSC. RCSC effects were better than Taxol® which showed only 30–50 % reduction of LDH at 96 h. Caco-2, the other cancer cell line did not show significant effect with most RCSC dilutions. Significant decrease in total LDH in normal colon cell line, NCM-356, was observed mostly at 72 h and 96 h, which was less than the effect of Taxol®. Scanning electron microscopy analysis of RCSC 1:5 treated SW620 showed reduced cell number, morphology and increased adhesiveness suggesting apoptosis (Fig. 1). These results suggest that lower dilutions (1:5 to 1:20) of RCSC would be better for further testing to treat colon cancer with minimal effect on normal mucosal cells.

RCSC showed significant effect on the membrane integrity of SW620 cells with leakage of LDH starting at 48 h. Cell density of 25,000 cells/mL showed the best results with 1:40 dilution (0.5 mg/mL) of RCSC showing maximum release of LDH at 72 h (Additional file 2: Figure S2). In Caco-2, lower dilutions of RCSC showed better results compared to higher dilutions. However, LDH release was less in Caco-2 cell line. No significant effect of RCSC on membrane integrity of the normal colon mucosal cell, NCM 356 was observed, which augurs well for the use of RCSC on animal model with the final goal to treat colon cancer.

WST-1 assay measures the metabolic viability of the cell through the reduction of tetrazolium salts by various mitochondrial reductases including LDH, which is a crucial metabolic enzyme in a cancer cell. RCSC treated SW620 showed a highly significant reduction in cell viability at 96 h (Additional file 3: Figure S3). In comparison, Taxol® reduced cell viability to a lesser extent than RCSC. In Caco-2 and NCM 356, a significant reduction was not observed at all RCSC dilutions and all time points tested.

#### Hormone independent breast cancer cell line responded better to RCSC treatment compared to hormone dependent breast cancer cell line

RCSC at 1:5 dilution showed the best effect in the hormone independent breast cancer cell line, MDA-MB 231 but other lower dilutions (1:10 and 1:20) were also very effective (Additional file 4: Figure S4). Taxol® was less effective than RCSC at 72 h and 96 h when compared to most RCSC dilutions. The effect of RCSC was more profound in 25,000 cells/mL compared 10,000 cells/mL of MDA-MB-231. The second breast cancer cell line, MCF-7, which is hormone dependent did not show a significant reduction in total LDH with RCSC treatment. Overall, MDA-MB-231 showed better response to RCSC than MCF-7. RCSC did not reduce the total LDH of the normal breast epithelial cell line, HMEC.

RCSC showed a significant effect in disrupting membrane integrity of the breast cancer cell line, MDA-MB-231 with higher dilutions of RCSC at 72 h (Additional file 5: Figure S5). The effect was comparatively less in MCF-7. RCSC also reduced the membrane integrity of normal breast epithelial cells (HMEC) with a significant release of LDH at all dilutions and treatment points (Additional file 6: Figure S6). LDH release gradually increased from 24 to 48 h with 221 % release of LDH in 1:5 dilution RCSC treatment followed by a decrease at 72 h and 96 h compared to 48 h. From these observations, RCSC had some effect in hampering the membrane integrity of MDA-MB-231 and MCF-7. However, a significant detrimental effect on the membranes of normal breast cells makes RCSC inefficient in treating breast cancer.

RCSC reduced cell viability based on WST-1 assay in MDA-MB-231 with lower RCSC dilutions showing maximum effect at 72 h and 96 h with 1:5 to 1:20 dilutions (Additional file 7: Figure S7). Effect of Taxol® was comparatively less than RCSC. RCSC did not show significant effect on the cell viability of MCF-7 and HMEC.

#### Lung cancer cell lines showed the best response to RCSC treatment

**Fig. 2:**
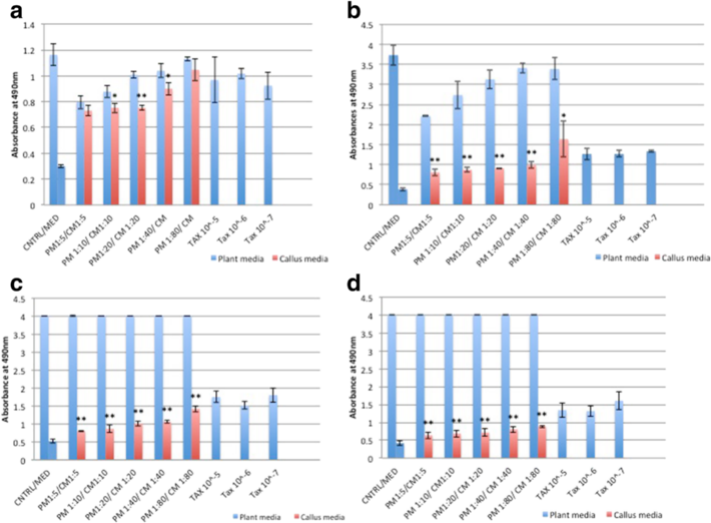
Total LDH of the lung cancer cell line, NCI-H460 treated with Rice Callus Suspension Culture (RCSC). Total LDH of NCI-H460 treated with different dilutions of rice callus suspension culture for (**a**) 24 h, (**b**) 48 h, (**c**) 72 h, and (**d**) 96 h. The x-axis shows five dilutions of plant media (PM), rice callus suspension culture media (CM) and three concentrations of Taxol®. The y-axis shows average absorbance at 490 nm for LDH assay. *Error bars* represent standard deviation. Statistically significant effect of different dilutions of CM is indicated by *p < 0.05 and **p < 0.01. Control represents untreated cell line and Med represents media without cells. Cell density of 25,000 cells/mL was used

**Fig. 3:**
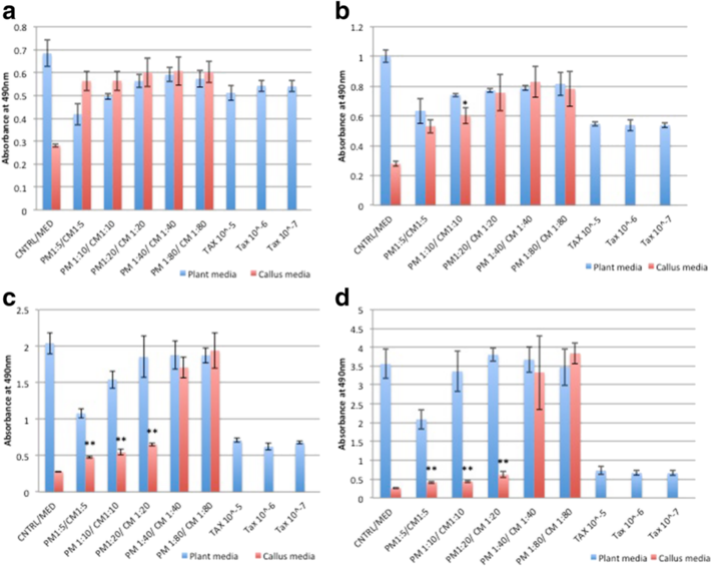
Total LDH of the lung cancer cell line, A-549 treated with RCSC. Total LDH of A-549 treated with different dilutions of rice callus suspension culture for (**a**) 24 h, (**b**) 48 h, (**c**) 72 h, and (**d**) 96 h. The x-axis shows five dilutions of plant media (PM), rice callus suspension culture media (CM) and three concentrations of Taxol®. Cell density used was 10,000 cells/mL. Labeling and statistical significance are as described for Fig. 1

RCSC significantly decreased LDH levels in NCI-H460 (non-small lung cancer cell line) and A549 cell line. RCSC effect on lung cancer cell lines was superior to the effect observed on colon and breast cancer cell lines. Lower dilutions of RCSC showed a better effect in reducing LDH than higher dilutions in NCI-H460 (Fig. 2). RCSC showed a significant effect at all treatment points and most dilutions of more than 95 % reduction in LDH with 1:5 to 1:80 dilution at 96 h in 25,000 cells/mL. On the other hand, A549 showed a better response at lower cell density (10,000 cells/mL) with lower dilutions (1:5, 1:10 and 1:20) of RCSC at 72 h and 96 h showing good results with a reduction in LDH levels reaching more than 95 % at 96 h with 1:5 dilution (Fig. 3). Both the lung cancer cell lines showed best effect at 1:5 dilution at 96 h. Effect of RCSC on NCI-H460 was better than Taxol® whereas its effect was comparable to Taxol® in A549 cell line. In normal lung fibroblast cell line, MRC-5, lower dilutions (1:5, 1:10 and 1:20) of RCSC showed significant reduction in LDH at 72 h at higher cell density (Additional file 8: Figure S8). Higher dilutions like 1:40 and 1:80 of RCSC showed less effect on MRC-5 whereas comparatively Taxol® showed more effect. These results showed less toxic effect of RCSC than cancer drugs such as Taxol®.

**Fig. 4:**
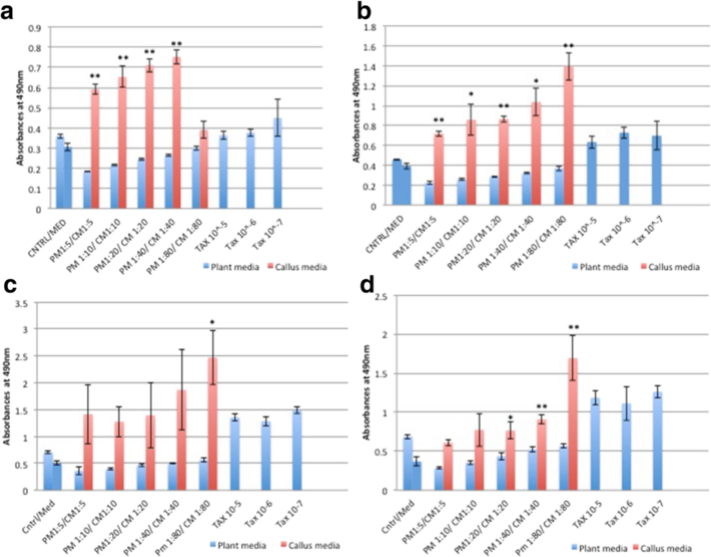
LDH released from the lung cancer cell line, NCI-H460 treated with RCSC. Release of LDH due to loss of membrane integrity of NCI-H460 treated with different dilutions of rice callus suspension culture for (**a**) 24 h, (**b**) 48 h, (**c**) 72 h, and (**d**) 96 h. Cell density used, labeling and statistical significance are as described for Fig. 1

Significant release of LDH due to loss of membrane integrity in treated NCI-H460 started at 24 h. The percentage of LDH released was the highest in RCSC 1:80 dilution treated sample in comparison to plant medium control (Fig. 4). The LDH released by Taxol® at all time points was lower than the highest release by RCSC treated sample. The membrane integrity was affected in A549 (25,000 cells/mL) on treatment with RCSC with maximum LDH release of 188 % at RCSC 1:10 dilution at 72 h. At 96 h, both NCI-H460 (147 %) and A549 (113 %) released less LDH compared to 72 h probably because of less total LDH content of the cell at 96 h as described earlier. The effect of RCSC on both NCI-H460 and A549 at lower cell density was less pronounced compared to their higher cell density. RCSC had no effect in disintegrating the membrane integrity of normal lung fibroblast MRC-5 at any dilution, time point and cell density. Lung cancer cell lines showed a better effect on disintegrating the membrane with the highest release of LDH (250 %) compared to colon and breast cancer cell lines. In conclusion, RCSC showed a superior effect on the lung cancer cell line, NCI-H460. It is likely that RCSC will be effective in treating lung cancer when a higher dilution like 1:40 or 1:80 is used where the reduction of LDH at higher cell density was calculated to be 73 % in NCI-H460 and 17 % in A549 at 48 h and the effect on MRC-5 was minimal.

**Fig. 5:**
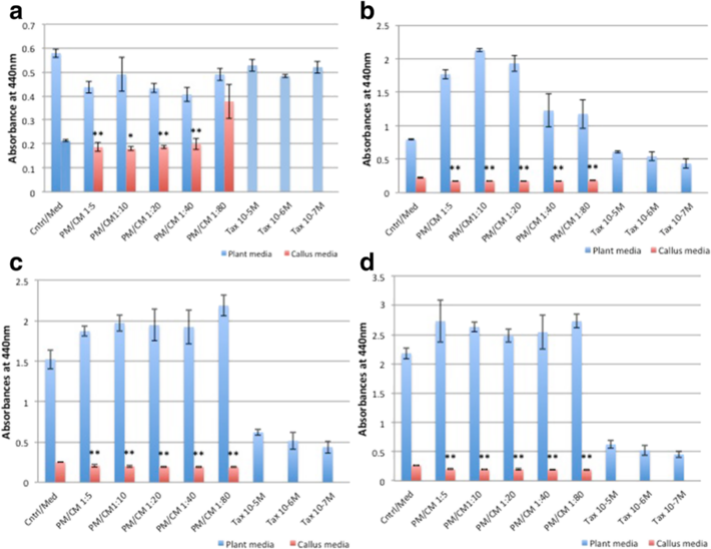
Cell viability of the lung cancer cell line, NCI-H460 treated with RCSC. Reduction of cell viability of NCI-H460 treated with different dilutions of RCSC for (**a**) 24 h, (**b**) 48 h, (**c**) 72 h, and (**d**) 96 h. The y-axis shows average absorbance at 440 nm for WST-1 assay. Cell density used, other labeling and statistical significance are as described for Fig. 1

RCSC hampered the metabolic capacity of NCI-H460 better than colon and breast cancer cell lines. RCSC effect on cell viability started at 24 h reaching maximal decrease (~95 %) with all RCSC dilutions at 96 h based on WST-1 assay (Fig. 5). Effect of Taxol® was comparatively lower than RCSC treatment at any time point and dilution. In A549, the effect of RCSC was less compared to NCI- H460. Cell viability in A549 was significantly reduced starting at 72 h and showed the best effect after 96 h at 1:5 and 1:10 RCSC dilutions (Additional file 9: Figure S9). Higher dilutions (1:20–1:80) did not show any effect on A549. Effects of Taxol® on A549 were less pronounced than RCSC treatment. In MRC-5, most RCSC dilutions and time points tested, did not show a significant reduction in cell viability.

It is evident that the lung cancer cell line, NCI-H460 was responding better to RCSC when compared to other tested cancer cell lines. To make an efficient anti-cancer agent, it is important to focus on minimal effect on normal cells. According to our observations, with reference to the lung cancer cell line, higher RCSC dilutions like 1:40 and 1:80 (i.e. 0.5 mg/mL and 0.25 mg/mL) showed less effect in lung fibroblast whereas in breast cancer 1:5 dilution (i.e. 4 mg/mL) of RCSC is better for treating breast cancer cell line since it showed minimal effect on human mammary epithelial cell, HMEC. In colon cancer, consistent effect of RCSC was measured at lower dilutions in SW620 with minimal or no effect on the normal colon mucosal cells and non-metastatic cancer cell line, Caco-2. In all of the above experiments, DMSO control (negative control) showed no effect on any of the cell lines (data not shown).

### RCSC treatment induced apoptosis in lung and colon cancer cell lines

Lung cancer cell line NCI-H460, which showed the best response with RCSC was chosen to further investigate the cellular and morphological changes in RCSC 1:5 and 1:40 dilution treated samples for 24 h and 72 h. RCSC treated NCI-H460 showed varying degrees of staining with signs of apoptotic changes. At 24 h, NCI-H460 treated with 1:5 dilution of RCSC showed clear signs of early stages of apoptosis process with nuclear blebbing and beginning of chromatin fragmentation in some cells (Additional file 10: Figure S10), which was accompanied by an increase in total fluorescence signal. On the other hand, PM stain showed unequal staining of cell membrane in RCSC 1:5 dilution treated NCI-H460 at 24 h and 72 h suggestive of loss of membrane structure, which also correlated with LDH release at that time point. Cell membrane in NCI-H460 was observed to be disintegrated in 1:40 dilution RCSC treatment at 72 h.

NucBlue staining was also performed on 25,000 cell density of MRC-5 treated with 1:5 and 1:40 dilution of RCSC. No significant changes in the nucleus or chromatin were observed. On the other hand, the colon cancer cell line, SW620, which gave good biochemical assay results with RCSC, showed some nuclear condensation and blebbing with 1:5 and 1:40 dilution RCSC for 72 h whereas PM stain showed undefined cell membrane with 1:5 dilution RCSC and well-defined cell membrane at 1:40 dilution RCSC treated sample and control (Additional file 11: Figure S11).

**Fig. 6:**
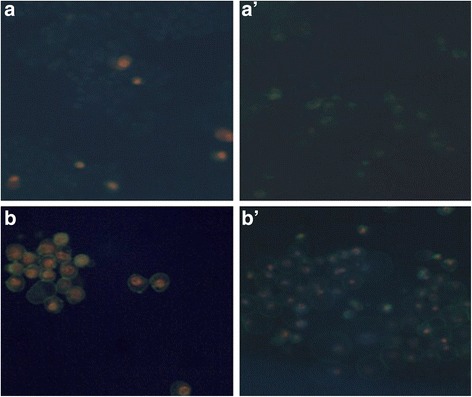
Annexin and propidium iodide (PI) staining of NCI-H460 treated with RCSC. (**a**) Control (untreated) at 24 h, (**b**) 1:5 dilution RCSC treated NCI-H460 at 24 h, (**a’**) Control at 72 h, (**b’**) 1:5 RCSC treated NCI-H460 at 72 h. NCI-H460 was labeled with Annexin and PI stain and observed at 365 nm (20× magnification)

NCI-H460 showed positive staining with annexin V and PI stain with 1:5 dilution RCSC at 24 h and 72 h compared to the control suggestive of late stage of apoptosis (Fig. 6). The cells showed positive PI stain due to RCSC affecting the membrane integrity as described earlier. RCSC 1:40 dilution showed less number of cells showing positive annexin V stain compared to 1:5 dilution of RCSC. Apoptosis was also observed in untreated cells (control) to a less extent suggesting that normal cell death mechanism was taking place.

Treatment of SW620 with RCSC 1:5 and 1:40 dilutions for 24 h were positive for PI staining but stained green (annexin V^+^) at 72 h suggestive of early stage of apoptosis process taking place (Additional file 12: Figure S12). In comparison to NCI-H460, both the stains were observed to be less in SW620. Based on annexinV and PI staining, it can be concluded that RCSC had significant effect on NCI-H460 at 24 h showing signs of late apoptosis whereas the effect on SW620 was observed later at 72 h.

**Fig. 7:**
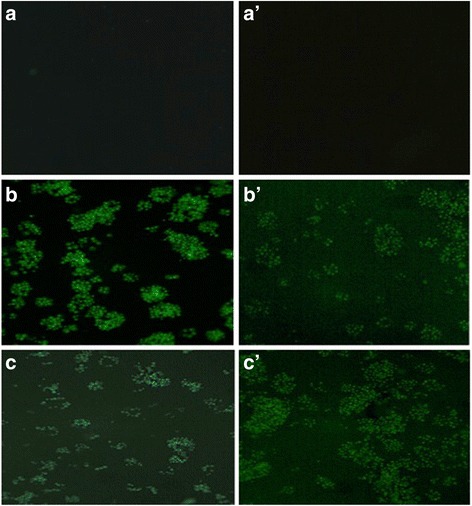
Caspase 3/7 staining of NCI-H460 treated with RCSC. (**a**) Control (untreated NCI-H460) at 24 h. (**b**) RCSC 1:5 dilution treatment at 24 h, (**c**) RCSC 1:40 dilution treatment at 24 h. (**a’**) Control at 72 h, (**b’**) RCSC 1:5 dilution treatment at 72 h, (**c’**) RCSC 1:40 dilution treatment at 72 h. Caspase 3/7 staining was observed at 470 nm (10× magnification)

Positive Caspase 3,7 activation was observed in both RCSC 1:5 and 1:40 dilution treated NCI-H460 at 24 h and 72 h (Fig. 7). Caspase activation was higher in 1:5 RCSC dilution treatment at 24 h compared to 72 h whereas 1:40 RCSC dilution treatment at 72 h showed higher fluorescence suggestive of ongoing apoptosis process. The corresponding cells demised at 1:5 RCSC dilution treatment at 72 h as observed in WST-1 and LDH assays. These observations were supported by fluorescence intensity based on Image J data (Additional file 13: Figure S13 and Additional file 14: Figure S14).

### RCSC treatment altered expression of genes involved in apoptotic pathways

**Fig. 8:**
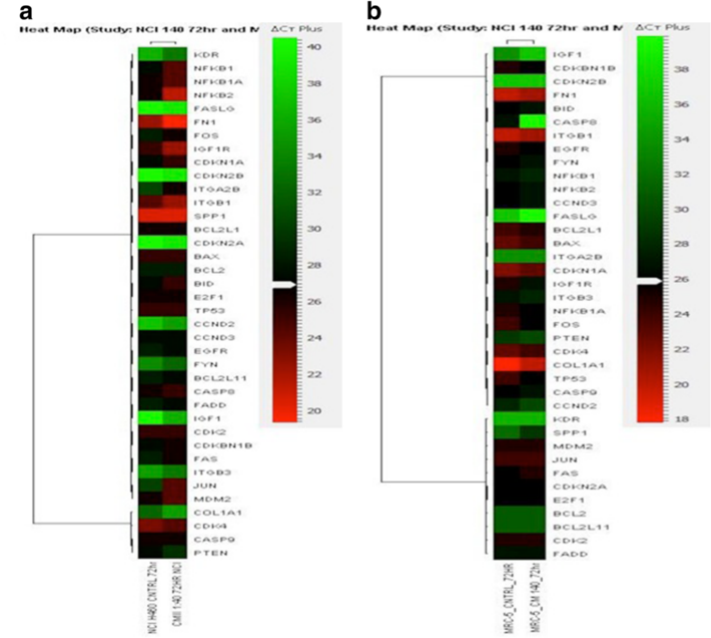
Heat map showing expression levels of selected genes in NCI-H460 treated with 1: 40 dilution of RCSC for 72 h. (**a**) NCI-H460 and (**b**) MRC-5. The ΔCt was compared with both their untreated controls. *Red color* represents upregulation and green color downregulation. Heat map was generated using Pearson correlation

Changes in expression of 92 genes involved in molecular mechanisms of cancer were investigated in NCI-H460 treated with RCSC to help us identify pathways that may be involved in maintaining its anticancer activity. Gradual increase in expression of cJUN, a proto-oncogene was observed from 24 h to 72 h with the highest expression of 61-fold upregulation in 1:40 RCSC dilution treated sample compared to the untreated control and negative control (Fig. 8a). CDKN1A, a gene, which codes for a cyclin-dependent kinase inhibitor, was upregulated 2.5 fold compared to the control. Genes downregulated compared to controls at 24 h on treatment with 1:5 RCSC dilution include insulin-like growth factor (IGF1) (10 fold), CyclinD_3_ (CCND3) (8 fold), E2 transcription factor 1 (E2F1) (8 fold), collagen type1 alpha 1 (COL1A1) (5 fold), and cyclin dependent kinase (CDK) (2.5 fold) (Additional file 15: Figure S15). FASLG gene was found to be 14-fold upregulated in 1:5 dilution RCSC treatment after 72 h. Nuclear Factor of Kappa light polypeptide gene enhancer in B-cells 2 (NF-κB2) gene was 2 fold and 30 fold upregulated in 1:5 and 1:40 RCSC dilution treated samples, respectively, at 72 h. Integrin α_2_ β (ITGA2B) and Integrin β_3_ (ITGB3) were upregulated 7 fold and 31 fold, respectively. Gene expression analysis of MRC-5 treated with 1:40 dilution RCSC for 72 h was performed to compare it with NCI-H460. No changes in gene expression were observed in MRC-5 compared to those observed in NCI-H460 suggesting that 1:40 dilution RCSC has no deleterious effects on MRC-5 (Fig. 8b).

### HPLC fractions of RCSC showed lower anti-cancer activity compared to whole RCSC

Lung cancer cell line, NCI-H460, which showed the best response to RCSC, was chosen to investigate the effects of four HPLC fractions of RCSC and plant medium. The fractions collected were calculated as a percentage of the total area of peaks at 254 nm. The selected fractions, 1, 2, 3 and 5 had 6.5 %, 37 %, 4 % and 6 % of the total peak area, respectively. The corresponding fractions were collected from plant medium and used as control (Additional file 16: Figure S16). No significant reduction of LDH was observed in the fraction treated samples compared to the whole RCSC treatment. Only 1:160 dilution of HPLC fraction 5 treated NCI-H460 (72 h) showed some reduction in total LDH (Additional file 17: Figure S17).

## Discussion

Cancer results from activation of oncogenes and/or suppression of tumor suppressor genes leading to important alterations in several signaling and metabolic pathways that are advantageous to cancer cell proliferation, survival, and progression. Altered metabolic pathways are very crucial for cancer cell proliferation and progression. Therefore, targeting metabolic pathways serves as a useful therapeutic strategy. Various plant extracts have been successful in altering the metabolic pathways resulting in the inhibition in cancer growth and progression. Our previous study showed that RCSC treatment resulted in significant reduction in cell viability in a single cell line of colon and renal cancer based on Crystal Violet assay [12]. In the present study, RCSC treatment showed a significant reduction in total LDH in cancer cells in a dose and time-dependent manner. LDH is a crucial cytosolic enzyme in cancer’s altered metabolism that is required for the proliferation and survivability of cancer cells. It is reported to be expressed at higher levels in cancer cells, due to alteration of various oncogenes or due to the overall tumor environment. Due to the hypoxic microenvironment in a rapidly proliferating cancer cell, LDH is essential to drive the rapid energy requirement of the cell through glycolytic metabolism. Glycolytic mechanism even though yields less energy, it provides energy faster, which is needed for a rapidly growing tumor cell. LDH converts pyruvate to lactate with the release of NAD^+^ that is required for the glycolytic pathway. It also helps in providing substrates for the synthesis of macromolecules required for the rapidly growing cell. The lactate produced by LDH has also been reported to have proliferating and migratory effects in cancer cells [16]. Various LDH knockdown experiments have been reported to repress tumor progression [17]. Prominent reduction in LDH due to RCSC may have halted the metabolism of the cell that resulted in inhibition of proliferation and possible induction of apoptosis. This inhibition was reported to occur by p53 dependent or independent pathways [18]. Inhibition of LDH may have diverted the cell to perform oxidative phosphorylation to produce energy that leads to excessive production of ROS causing detrimental effects to the cell. Increase in ROS degrades DNA, macromolecules and activates cell death mechanism [19]. This could be one possible reason as to why we observed cell death process in RCSC treated cancer cells. The increase in ROS also has membrane-degrading activities affecting mitochondrial membrane integrity and trans-membrane potential resulting in induction of intrinsic pathway of apoptosis. This is most likely the reason for higher release of LDH in non-lysed cells. RCSC probably impaired cell membrane of lung and colon cancer cell lines in a dose dependent manner either indirectly through ROS or directly. Cell membrane is the integral part of any cell, which helps to maintain homeostasis of the cell and also regulates many vital characteristics of the cell such as cell to cell adhesion, metastasis, changes in antigenicity and various surface proteins. Release of LDH or uptake of dyes like PI or trypan blue is a proxy for the loss of membrane integrity. Loss of membrane integrity always correlates with loss of viability and has been reported to induce cell death [20]. In the current study, LDH showed a maximum release at 72 h, which correlated with cell mask PM staining of NCI-H460 and SW620 accompanied with changes in cell membrane morphology. Cell membranes of cancer cells are always in a constant stress due to its physiology and the surrounding environment and undergo a continuous repair through the plasma membrane repair mechanism (PMR) to maintain and stabilize its integrity [21]. This might be a possible reason for the decrease in LDH release at 96 h compared to 72 h in cancer cell lines. The other possibility could be an overall decrease in LDH content of the cell due to the effect of RCSC as maximum reduction in total LDH was observed at 96 h. A concentrated RCSC solution (1:5 dilution) released less LDH than a diluted RCSC solution (1:80), which can be correlated with the total reduction of LDH at those particular dilutions. Loss of membrane integrity also explains why PI^+^ staining was observed on treatment with RCSC.

Early and late signs of apoptotic changes were clearly observed in fluorescence microscopy indicating the processes induced by RCSC. Apoptosis and necrosis are two different types of cell death that can happen simultaneously especially in the early stages. This was observed at 1:5 dilution treated NCI-H460 at 24 and 72 h. Apoptosis is characterized by shrinkage of the cell, nucleus, and chromatin condensation and finally formation of apoptotic bodies. Necrosis is characterized by cell swelling with rupture of the membrane and expulsion of the cell organelles and components. One basic difference between apoptosis and necrosis is the intact cell membrane seen in apoptotic cell death, which can be identified by the PI stain, a red non-permeable stain that only enters cell when the cell membrane is disintegrated. Under inverted light microscope, the cell looked pyknotic and granular suggestive of apoptotic bodies. Another indicator of the apoptotic process is positive caspase 3, 7 staining. Caspases are a family of enzymes, which are cysteine aspartic acid proteases mainly involved in the initiation, and execution of apoptosis. Positive caspase 3 and 7 staining was observed in RCSC treated NCI-H460 indicating activation of caspase 3 and 7, implying that execution process of apoptosis is in play, which is characterized by cell shrinkage (pyknosis), chromatin condensation followed by nuclear condensation and fragmentation. This is followed by phagocytosis by macrophages to complete the cell death mechanism in vivo [20]. The gene encoding FASLG protein, which belongs to the tumor necrosis factor superfamily, was upregulated in NCI-H460 treated with RCSC for 72 h. FASLG is involved in the extrinsic pathway of apoptosis and is reported to be induced by NF-κB cascade. Upregulation of this gene also increases T cell and cytotoxic T lymphocyte induced cell death [22]. It is possible that the effect on the cell membrane or targeting and reducing LDH may have upregulated FASLG initiating the apoptotic process.

Gene expression studies identified changes in potential cancer inhibitory protein encoding genes in RCSC treated lung cancer cell line, NCI-H460. In comparison, no such changes were observed in the normal cell line, MRC-5. Upregulation of genes involved in cell cycle inhibition, induction of apoptosis either directly or indirectly was observed. Expression of cJUN, a proto-oncogene which is a component of activator protein (AP1) family [23] related to the PI3K-AKT and MAPK pathway, was upregulated. P13K-AKT pathway is an important metabolic pathway modulator of tumor cells. It regulates and maintains the glycolytic phenotype of tumor cell through expression of various glycolytic enzymes, cell membrane glucose transporters, and stimulates other biosynthetic pathways required for the survival of tumorigenesis [24]. Recently, cJUN was found to have both tumor promotive and inhibitory effect. Over expression of cJUN was reported in cells arrested in G_1_ phase of cell cycle. Upregulation of cJUN was reported to downregulate the expression of various cyclin dependent kinases [23] as it competitively binds to the AP1 site in the cyclin gene promoter. cJUN has been also reported to induce apoptosis via the NF-κB-JNK signaling cascade. For example, grape extract has been reported to induce apoptosis in prostate cancer cells via Jun N kinase (JNK) signaling and cJUN activation [25]. JNK signaling is reported to activate cJUN by phosphorylation. Another upregulated gene was CDKN1A, a cyclin-dependent kinase inhibitor needed in maintaining the progression of the cell cycle. It is regulated by p53, a tumor suppressor and helps in upregulating the expression of various antioxidant and detoxifying agents [24].

Another group of genes that showed a significant upregulation on RCSC treatment were those encoding integrin α_2_ β (ITGA2B) and integrin β_3_ (ITGB3), which belong to the cell adhesion molecules of the integrin family, known to associate with the tyrosine kinase signaling pathways and regulate cell survival, proliferation, differentiation, adhesion, and migration [26]. They are known to have cell adhesive properties essential for metastasis. There are various types of integrins, namely, α_26_β1, α_v_ β5, α_3_ β1 whose expression increased in tumor cells compared to normal cells [27]. An exception to this pattern is reported for integrin α_2_ β (ITGA2B) and integrin β_3_ (ITGB3). Expression of ITGA2B was reported to be debilitating to some tumor cells such as breast cancer cells, which resulted in changing of the malignant phenotype to normal [28]. Similarly, upregulation of ITGA2B on treatment with RCSC probably suppressed NCI-H460 proliferation and survival. ITGB3, on the other hand, showed apoptosis inducing property in ovarian cancer, gliomas and melanoma [29, 30]. Unligated integrins form a complex with caspase 8 and activate the extrinsic pathways of apoptosis [31]. Here, in our in vitro experiment, integrins were probably involved in inducing apoptosis since integrins are unligated due to the absence of a ligating protein of the extracellular matrices that are present in vivo. These genes showed no change in expression in normal lung fibroblast, MRC-5 making RCSC an effective anticancer agent for lung cancer.

In addition to upregulation of genes, a number of cancer-promoting genes were downregulated. Downregulation of cyclin D3 (CCND3) and cyclin dependent kinase (CDK) indicates abrupt halt in cell cycle as the first line effect of RCSC on NCI-H460 at 24 h. This is supported by the upregulation of cJUN at 72 h in 1:5 RCSC treated NCI-H460 which suggests cell cycle arrest. E2F1 maintains a balance between tumor proliferative and tumor suppressive functions. In cancer cells, the PI3K-AKT pathway activates E2F1 thereby inactivating tumor suppressive activity [32]. RCSC treatment leads to downregulation of E2F1, which is conducive for anticancer treatment. COL1A1 codes a protein associated with platelet-derived growth factor (PDGF) signaling pathway, which helps in the maintenance of tumor stromal and angiogenesis facilitation in metastasis [33]. The downregulation of COL1A1 on RCSC treatment points to inhibition of cancer cell progression and possibility of angiogenesis. Cancer migration or metastasis is a crucial factor in any cancer prognosis. A metastatic cancer cell has been reported to be highly fatal with bad prognosis with any cancer treatment. It is important to note that RCSC is effectively preventing the metastatic power of the NCI-H460 and thus could serve as a potent agent in targeting cancer. IGF-1 is a mitogen, which is usually overexpressed in cancer cells maintaining cell cycle program regulated by the IGF-1R and help in transformation and protection of cancer from apoptosis [34]. IGF-1 was also found to make cancer cells resistant to apoptosis induced by anti-cancer drugs [35]. In this context, downregulation of IGF-1 is potentially beneficial in treating cancer by RCSC alone or in combination with a known anti-cancer drug.

**Fig. 9:**
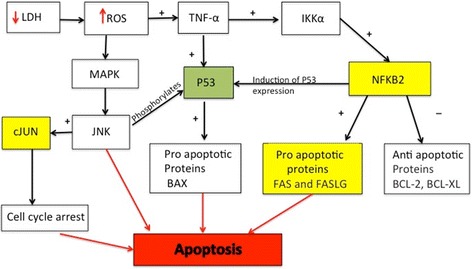
Putative pathways involved in inducing apoptosis on RCSC treatment. Proteins regulating apoptosis and encoded by genes upregulated on RCSC treatment are highlighted in *yellow boxes*

RCSC treatment upregulated genes that not only help in cancer promotion by regulating tumor proliferation, survival, progression, and metastasis but also have tumor suppressive activities. One such gene was NF-κB2, a transcription factor, which was upregulated in both 1:5 and 1:40 dilution RCSC treated NCI-H460 at 72 h with higher expression at 1:40 dilution. In addition to tumor promoting properties, NF-κB2 has been reported to have tumor suppressive activities including induction of apoptosis, which is mainly mediated by the tumor suppressor gene, p53 [36]. These cancer suppressive activities of NF-κB2 is dependent upon the cell type, the type of inhibitory stimulus, and the duration of stimulus or signals [37, 38]. DNA damage was found to activate their tumor suppressive effect through recruitment and activation of death receptor inducing the extrinsic pathway of apoptosis. It is also possible that the upregulation of NF-κB2 could be a cancer protective mechanism in response to RCSC treatment. The possible pathway leading to apoptosis mediated by RCSC treatment through upregulated genes in our study, is shown in Fig. 9.

Our final investigation involved studying the effect of RCSC fractions on LDH content of NCI-H460 at four time points, which indicated RCSC treatment to be more effective than its fractionated components. This supports the hypothesis that a holistic approach of using the whole RCSC with the synergistic effect of several compounds is more effective than purified compounds or fractions. The synergistic effect of multiple compounds is likely to eliminate or minimize the harmful effects of purified compounds on normal cells.

## Conclusions

RCSC was shown to be superior to Taxol® as it effectively inhibited proliferation of lung and colon cancer cells with minimal or less effect compared to normal cells in a dose and time dependent manner. However, it was less promising with breast cancer cells. A decrease in the total LDH content in cancer cell lines may have elicited the upregulation of genes inhibiting cell cycle and possibly metastatic and angiogenic capability thereby inducing apoptosis. Further investigation is necessary to identify the molecular basis of RCSC activity by using systems biology approach employing gene expression profiling, proteomics and metabolomics. Unlike several plant isolated compounds showing potent anticancer effect, RCSC fractions were found to be less effective than whole RCSC. Further investigations using other methods for isolation of RCSC compounds followed by in vitro treatment, can help in arriving at concrete conclusion. The current study showed positive results on the potency of RCSC, which have to be confirmed in animal models followed by clinical trials. It is likely that this will lead to the development of a better drug that is much more efficient with minimal side effect than the current anticancer drugs.

## Additional files

Additional file 1: Figure S1. Total LDH of the colon cancer cell line, SW620 treated with RCSC. Total LDH in SW620 treated with different dilutions of rice callus suspension culture for (A) 24 h, (B) 48 h, (C) 72 h, and (D) 96 h. Cell density used, labeling and statistical significance are as described for Fig. 1. (JPG 113 kb)
Additional file 2: Figure S2. LDH release from the colon cancer cell line, SW620 treated with RCSC. Release of LDH due to loss of membrane integrity of SW620 treated with different dilutions of RCSC for (A) 24 h, (B) 48 h, (C) 72 h, and (D) 96 h. Cell density used, labeling and statistical significance are as described for Fig. 1. (JPG 105 kb)
Additional file 3: Figure S3. Cell viability of the colon cancer cell line, SW620 treated with RCSC. Reduction of cell viability of SW620 treated with different dilutions of rice callus suspension culture for (A) 24 h, (B) 48 h, (C) 72 h, and (D) 96 h. The y-axis shows average absorbance at 440 nm for WST-1 assay. Cell density used, other labeling and statistical significance are as described for Fig. 1. (JPG 111 kb)
Additional file 4: Figure S4. Total LDH of the breast cancer cell line, MDA-MB-231 treated with RCSC. Total LDH in MDA-MB-231 treated with different dilutions of RCSC for (A) 24 h, (B) 48 h, (C) 72, and (D) 96 h. Cell density used, labeling and statistical significance are as described for Fig. 1. (JPG 109 kb)
Additional file 5: Figure S5. LDH release from the breast cancer cell line, MDA-MB-231 treated with RCSC. Release of LDH due to loss of membrane integrity of MDA-MB-231 treated with different dilutions of rice callus suspension culture for (A) 24 h, (B) 48 h, (C) 72 h, and (D) 96 h. Cell density used, labeling and statistical significance are as described for Fig. 1. (JPG 110 kb)
Additional file 6: Figure S6. LDH release from the breast epithelial cell line, HMEC treated with RCSC. Release of LDH due to loss of membrane integrity of HMEC treated with different dilutions of rice callus suspension culture for (A) 24 h, (B) 48 h, (C) 72, and (D) 96 h. Cell density used, labeling and statistical significance are as described for Fig. 1. (JPG 107 kb)
Additional file 7: Figure S7. Cell viability of the breast cancer cell line, MDA-MB-231 treated with RCSC. Reduction of cell viability of MDA-MB-231 treated with different dilutions of rice callus suspension culture for (A) 24 h, (B) 48 h, (C) 72 h, and (D) 96 h. The y-axis shows average absorbance at 440 nm for WST-1 assay. Cell density used, other labeling and statistical significance are as described for Fig. 1. (JPG 109 kb)
Additional file 8: Figure S8. Total LDH of the lung fibroblast cell line, MRC-5 treated with RCSC. Total LDH of MRC-5 treated with different dilutions of rice callus suspension culture for (A) 24 h, (B) 48 h, and (C) 72 h, and (D) 96 h. Cell density used, labeling and statistical significance are as described for Fig. 1. (JPG 115 kb)
Additional file 9: Figure S9. Cell viability of the lung cancer cell line, A-549 treated with RCSC followed by WST-1 assay. Reduction of cell viability of A-549 treated with different dilutions of rice callus suspension culture for (A) 24 h, (B) 48 h, (C) 72 h, and (D) 96 h. The y-axis shows average absorbance at 440 nm for WST-1 assay. Cell density used, other labeling and statistical significance are as described for Fig. 1. (JPG 108 kb)
Additional file 10: Figure S10. Nuclear and plasma membrane staining of NCI-H460 treated with RCSC. (A & A’) Control (untreated NCI-H460), (B & B’) Treated with 1:5 dilution RCSC and (C & C’) Treated with 1:40 dilution RCSC for 72 h. A, B and C represent NCI-H460 labeled with NucBlue live cell stain (365 nm) and A’, B’ and C’ represent cells labeled with cell mask plasma membrane stain (475 nm) observed at 20× magnification. (JPG 67 kb)
Additional file 11: Figure S11. Nuclear and plasma membrane staining of SW620 treated with RCSC for 72 h. (A & A’) Control at 72 h. (B & B’) RCSC 1:5 dilution treatment for 72 h. (C & C’) RCSC 1:40 dilution treatment for 72 h. SW620 was labeled with NucBlue live cell stain (365 nm) and cell mask plasma membrane stain (475 nm) and observed at 20× magnification. (JPG 65 kb)
Additional file 12: Figure S12. Annexin and PI staining of SW620 treated with RCSC. (A) Control (untreated) at 24 h, (B) 1:5 diluted RCSC treated at 24 h, (C) 1:40 diluted RCSC treated at 24 h. (A’) Control at 72 h, (B’) 1:5 diluted RCSC treated at 72 h and (C’) 1:40 diluted RCSC treated at 72 h. Annexin and PI stained cells were observed at 365 nm at 20× magnification. Apoptosis (Annexin^+^/PI^−^) was dominant at 72 h in both 1:40 and some 1:5 RCSC dilution treated SW620. 1:40 dilution treated SW620 at 24 h was mostly PI^+^. (JPG 29 kb)
Additional file 13: Figure S13. Bar diagram showing quantitative Caspase 3/7 fluorescence in NCI-H460 after RCSC treatment for 24 h. Image J analysis was used to quantify fluorescence microscope images which showed caspase activation in RCSC 1:5 and 1:40 dilution treated NCI-H460. CTCF represents Corrected Total Cell Fluorescence (n = 5). (PNG 127 kb)
Additional file 14: Figure S14 Bar diagram showing quantitative Caspase 3/7 fluorescence in NCI-H460 after RCSC treatment for 72 h. Image analysis and labeling are as described for Additional file 12: Figure S12. (JPG 34 kb)
Additional file 15: Figure S15. Heat map representing expression levels of genes associated with cancer in NCI-H460 treated with 1:5 dilution RCSC for 72 h. The ΔCt was compared with untreated control. The red bar represents upregulation and green downregulation. Heat map was generated using Pearson correlation. Callus and NCI CM 72 h represent samples treated with RCSC 1:5 dilution for 24 h and 72 h, respectively. (JPG 35 kb)
Additional file 16: Figure S16. HPLC chromatogram showing peaks at 254 nm. HPLC fractionation of RCSC showing peak heights on y-axis and retention time on x-axis. Fractions 1, 2, 3, and 5 selected for treatment of the lung cancer cell line are shown in the figure. (JPG 51 kb)
Additional file 17: Figure S17. Total LDH absorbance of NCI-H460 treated with RCSC and its HPLC fractions. Total LDH absorbance of NCI-H460 treated with five HPLC fractions of RCSC and plant media for (A) 24 h, (B) 48 h, (C) 72 h, and (D) 96 h. The x-axis shows different dilutions of plant media (PM), plant media fractions (PM fr.1, PM fr.2, PM fr. 3, PM fr.5), RCSC or callus media (CM) and callus media fractions (CM fr.1, CM fr.2, CM fr. 3, CM fr. 5). The y-axis shows average absorbance at 490 nm of LDH assay. Error bars represent standard deviation. Statistically significant effect of different dilutions of callus culture on the viability of cell lines is indicated by *p < 0.05 and **p < 0.01. Control represents untreated cell line and Med represents media without cells. (ZIP 185 kb)

## Abbreviations

2,4D: 2,4 dichlorophenoxyacetic acid
ATCC: American type for culture collection
CTCF: Corrected total cell fluorescence
DMEM: Dulbecco’s minimal essential medium
EMEM: Eagle’s minimum essential medium
FBS: Fetal bovine serum
HPLC: High performance liquid chromatography
LDH: Lactate dehydrogenase
MS: Murashige and Skoog
PM: Plant medium
RCSC: Rice callus suspension culture
WHO: World Health Organization
