# Supplementary figures and images for: Rice callus suspension culture inhibits growth of cell lines of multiple cancer types and induces apoptosis in lung cancer cell line
Source: BMC Complement Altern Med. 2016 Nov 2;16:427. doi: 10.1186/s12906-016-1423-3 (PMC5093976; doi:10.1186/s12906-016-1423-3)

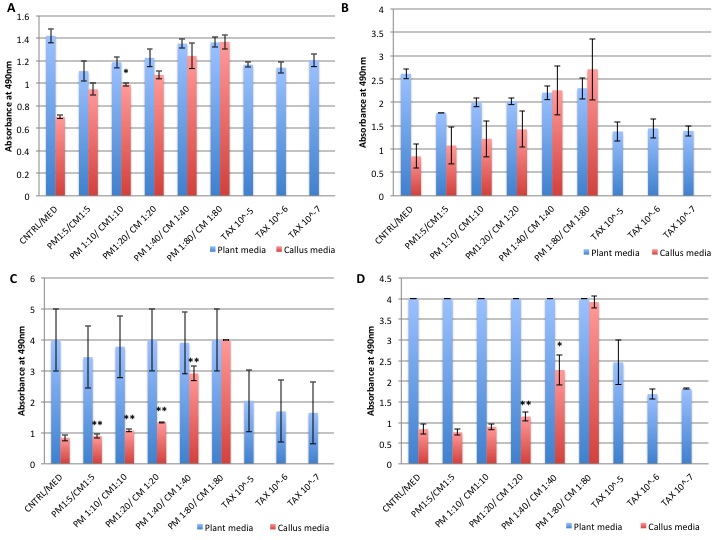

Supplement: Additional file 1: Figure S1. — Total LDH of the colon cancer cell line, SW620 treated with RCSC. Total LDH in SW620 treated with different dilutions of rice callus suspension culture for (A) 24 h, (B) 48 h, (C) 72 h, and (D) 96 h. Cell density used, labeling and statistical significance are as described for Fig. 1. (JPG 113 kb) [file 12906_2016_1423_MOESM1_ESM.jpg]

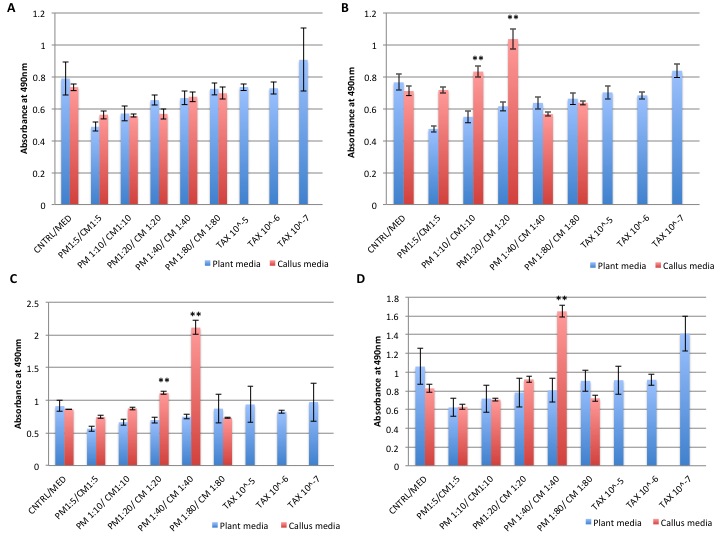

Supplement: Additional file 2: Figure S2. — LDH release from the colon cancer cell line, SW620 treated with RCSC. Release of LDH due to loss of membrane integrity of SW620 treated with different dilutions of RCSC for (A) 24 h, (B) 48 h, (C) 72 h, and (D) 96 h. Cell density used, labeling and statistical significance are as described for Fig. 1. (JPG 105 kb) [file 12906_2016_1423_MOESM2_ESM.jpg]

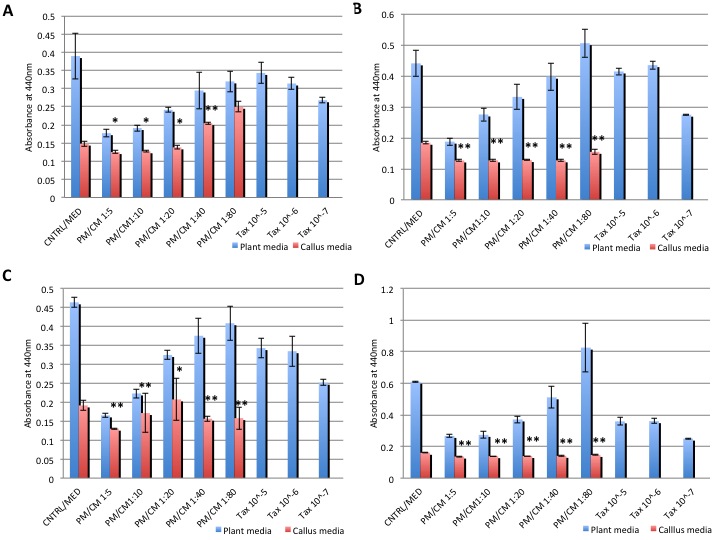

Supplement: Additional file 3: Figure S3. — Cell viability of the colon cancer cell line, SW620 treated with RCSC. Reduction of cell viability of SW620 treated with different dilutions of rice callus suspension culture for (A) 24 h, (B) 48 h, (C) 72 h, and (D) 96 h. The y-axis shows average absorbance at 440 nm for WST-1 assay. Cell density used, other labeling and statistical significance are as described for Fig. 1. (JPG 111 kb) [file 12906_2016_1423_MOESM3_ESM.jpg]

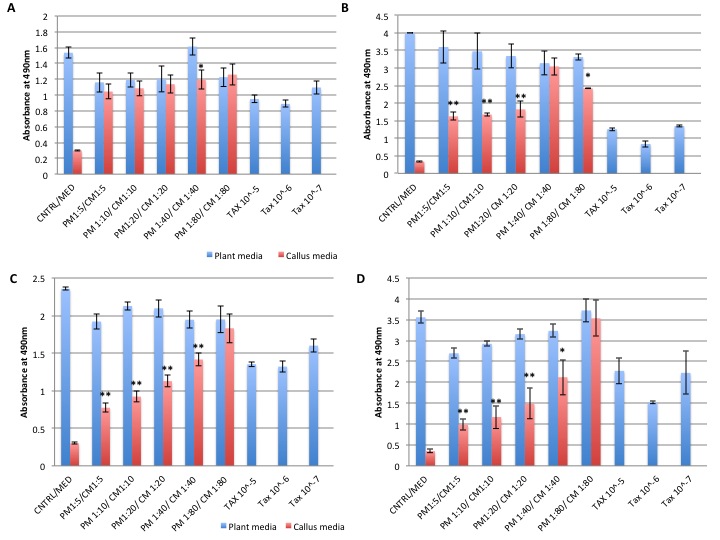

Supplement: Additional file 4: Figure S4. — Total LDH of the breast cancer cell line, MDA-MB-231 treated with RCSC. Total LDH in MDA-MB-231 treated with different dilutions of RCSC for (A) 24 h, (B) 48 h, (C) 72, and (D) 96 h. Cell density used, labeling and statistical significance are as described for Fig. 1. (JPG 109 kb) [file 12906_2016_1423_MOESM4_ESM.jpg]

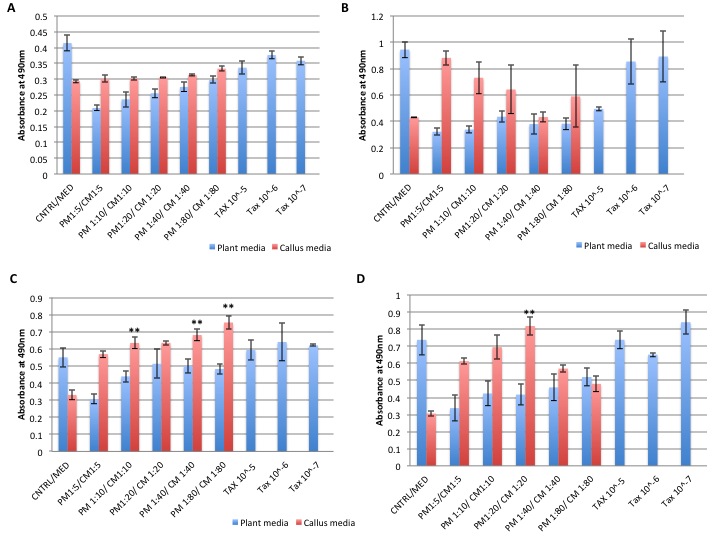

Supplement: Additional file 5: Figure S5. — LDH release from the breast cancer cell line, MDA-MB-231 treated with RCSC. Release of LDH due to loss of membrane integrity of MDA-MB-231 treated with different dilutions of rice callus suspension culture for (A) 24 h, (B) 48 h, (C) 72 h, and (D) 96 h. Cell density used, labeling and statistical significance are as described for Fig. 1. (JPG 110 kb) [file 12906_2016_1423_MOESM5_ESM.jpg]

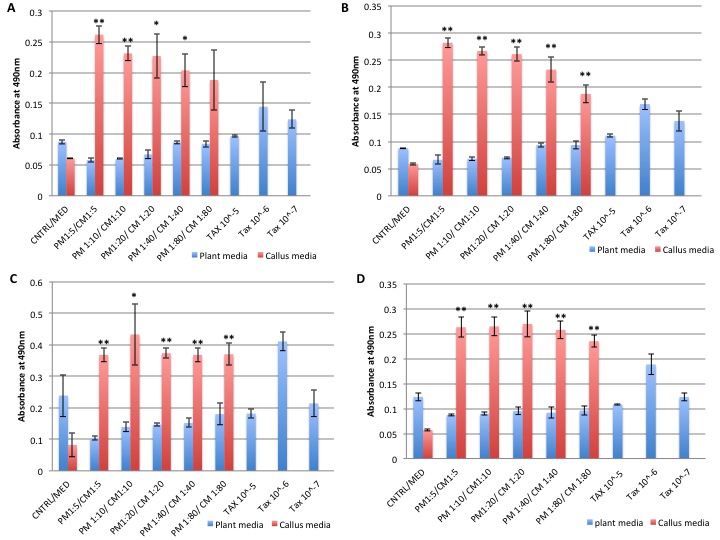

Supplement: Additional file 6: Figure S6. — LDH release from the breast epithelial cell line, HMEC treated with RCSC. Release of LDH due to loss of membrane integrity of HMEC treated with different dilutions of rice callus suspension culture for (A) 24 h, (B) 48 h, (C) 72, and (D) 96 h. Cell density used, labeling and statistical significance are as described for Fig. 1. (JPG 107 kb) [file 12906_2016_1423_MOESM6_ESM.jpg]

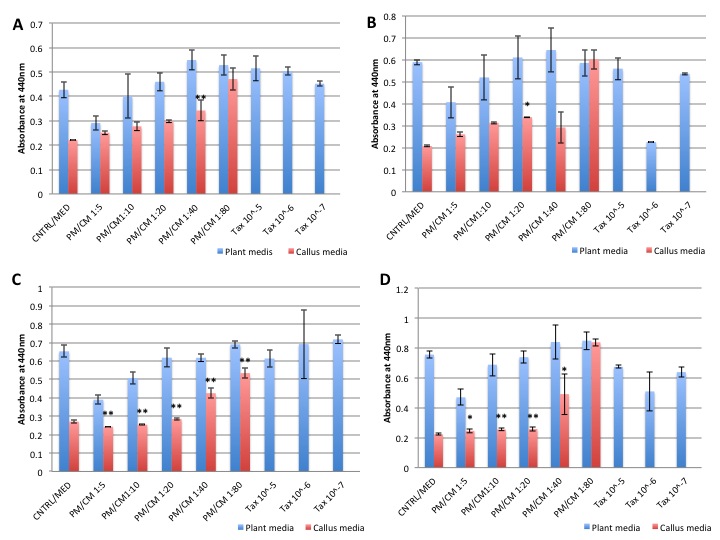

Supplement: Additional file 7: Figure S7. — Cell viability of the breast cancer cell line, MDA-MB-231 treated with RCSC. Reduction of cell viability of MDA-MB-231 treated with different dilutions of rice callus suspension culture for (A) 24 h, (B) 48 h, (C) 72 h, and (D) 96 h. The y-axis shows average absorbance at 440 nm for WST-1 assay. Cell density used, other labeling and statistical significance are as described for Fig. 1. (JPG 109 kb) [file 12906_2016_1423_MOESM7_ESM.jpg]

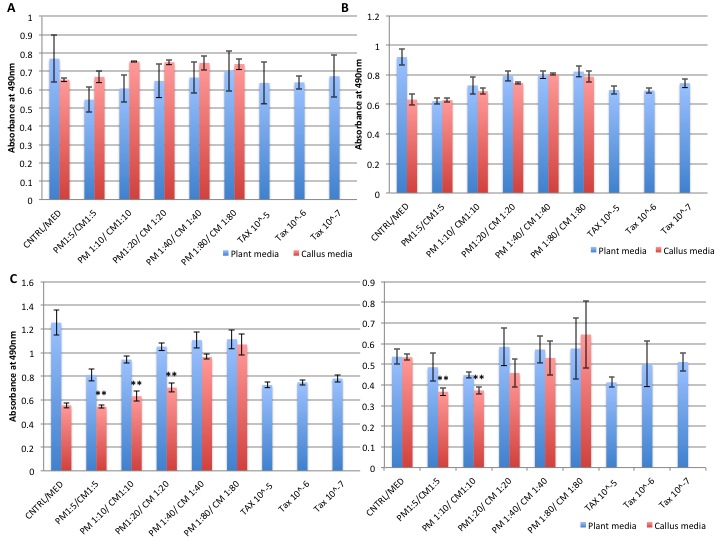

Supplement: Additional file 8: Figure S8. — Total LDH of the lung fibroblast cell line, MRC-5 treated with RCSC. Total LDH of MRC-5 treated with different dilutions of rice callus suspension culture for (A) 24 h, (B) 48 h, and (C) 72 h, and (D) 96 h. Cell density used, labeling and statistical significance are as described for Fig. 1. (JPG 115 kb) [file 12906_2016_1423_MOESM8_ESM.jpg]

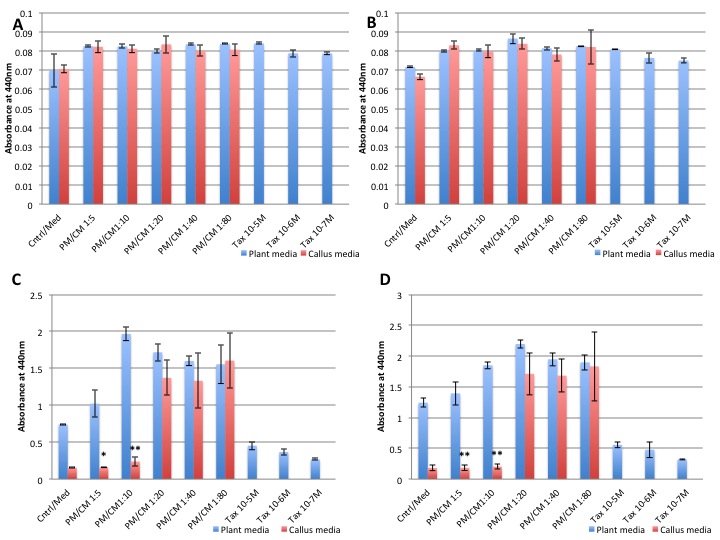

Supplement: Additional file 9: Figure S9. — Cell viability of the lung cancer cell line, A-549 treated with RCSC followed by WST-1 assay. Reduction of cell viability of A-549 treated with different dilutions of rice callus suspension culture for (A) 24 h, (B) 48 h, (C) 72 h, and (D) 96 h. The y-axis shows average absorbance at 440 nm for WST-1 assay. Cell density used, other labeling and statistical significance are as described for Fig. 1. (JPG 108 kb) [file 12906_2016_1423_MOESM9_ESM.jpg]

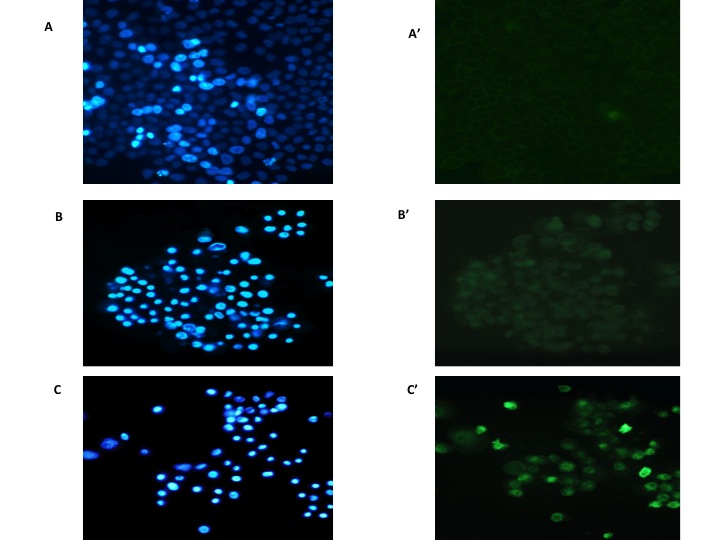

Supplement: Additional file 10: Figure S10. — Nuclear and plasma membrane staining of NCI-H460 treated with RCSC. (A & A’) Control (untreated NCI-H460), (B & B’) Treated with 1:5 dilution RCSC and (C & C’) Treated with 1:40 dilution RCSC for 72 h. A, B and C represent NCI-H460 labeled with NucBlue live cell stain (365 nm) and A’, B’ and C’ represent cells labeled with cell mask plasma membrane stain (475 nm) observed at 20× magnification. (JPG 67 kb) [file 12906_2016_1423_MOESM10_ESM.jpg]

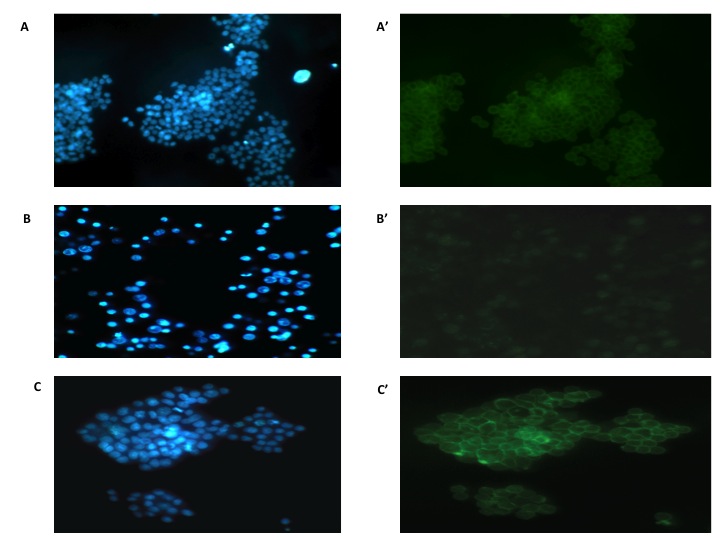

Supplement: Additional file 11: Figure S11. — Nuclear and plasma membrane staining of SW620 treated with RCSC for 72 h. (A & A’) Control at 72 h. (B & B’) RCSC 1:5 dilution treatment for 72 h. (C & C’) RCSC 1:40 dilution treatment for 72 h. SW620 was labeled with NucBlue live cell stain (365 nm) and cell mask plasma membrane stain (475 nm) and observed at 20× magnification. (JPG 65 kb) [file 12906_2016_1423_MOESM11_ESM.jpg]

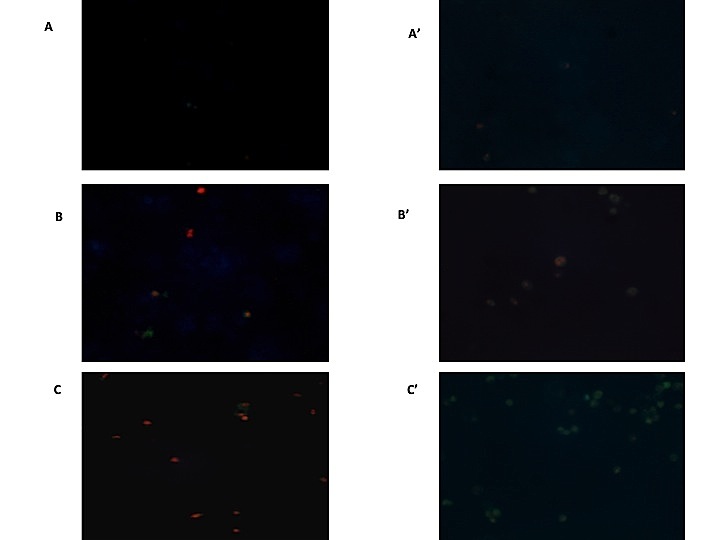

Supplement: Additional file 12: Figure S12. — Annexin and PI staining of SW620 treated with RCSC. (A) Control (untreated) at 24 h, (B) 1:5 diluted RCSC treated at 24 h, (C) 1:40 diluted RCSC treated at 24 h. (A’) Control at 72 h, (B’) 1:5 diluted RCSC treated at 72 h and (C’) 1:40 diluted RCSC treated at 72 h. Annexin and PI stained cells were observed at 365 nm at 20× magnification. Apoptosis (Annexin+/PI−) was dominant at 72 h in both 1:40 and some 1:5 RCSC dilution treated SW620. 1:40 dilution treated SW620 at 24 h was mostly PI+. (JPG 29 kb) [file 12906_2016_1423_MOESM12_ESM.jpg]

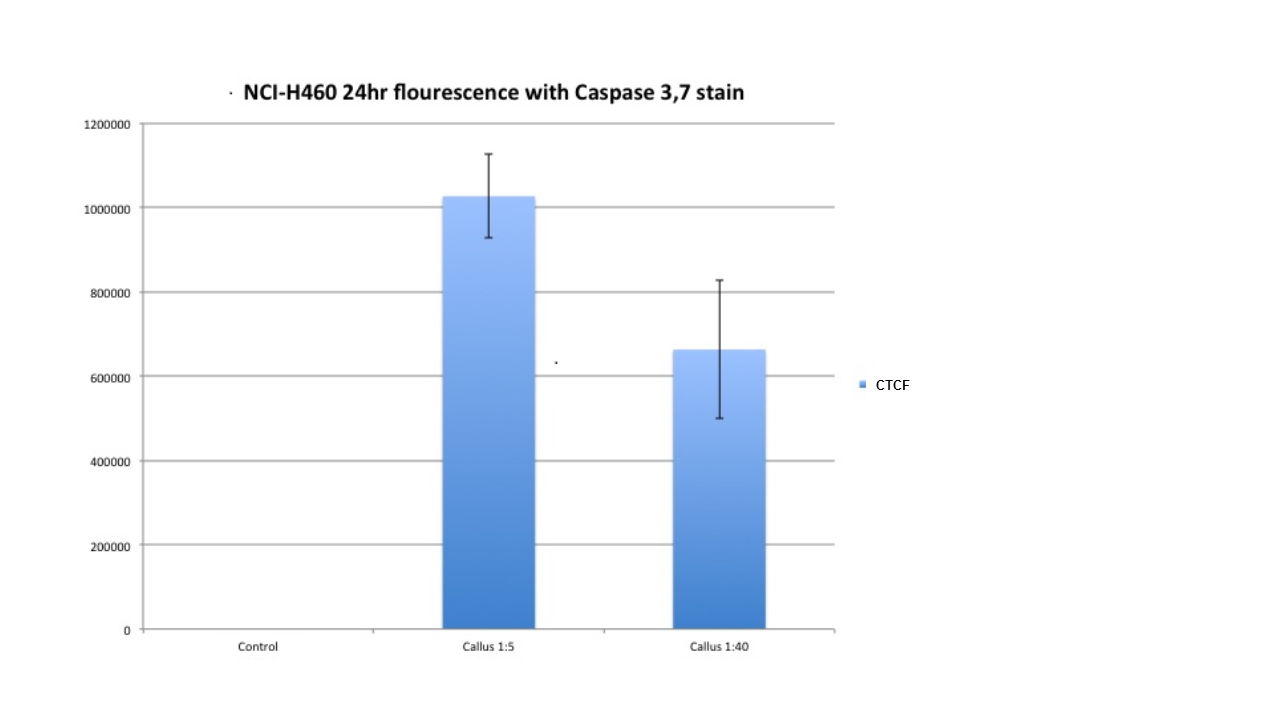

Supplement: Additional file 13: Figure S13. — Bar diagram showing quantitative Caspase 3/7 fluorescence in NCI-H460 after RCSC treatment for 24 h. Image J analysis was used to quantify fluorescence microscope images which showed caspase activation in RCSC 1:5 and 1:40 dilution treated NCI-H460. CTCF represents Corrected Total Cell Fluorescence (n = 5). (PNG 127 kb) [file 12906_2016_1423_MOESM13_ESM.png]

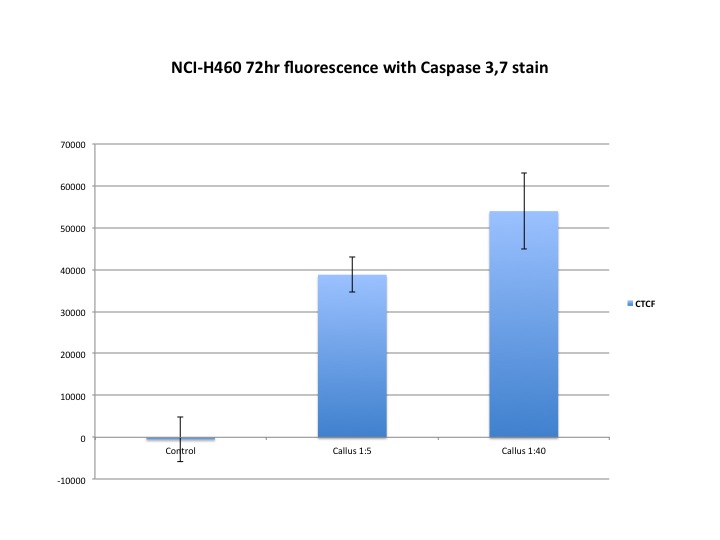

Supplement: Additional file 14: Figure S14 — Bar diagram showing quantitative Caspase 3/7 fluorescence in NCI-H460 after RCSC treatment for 72 h. Image analysis and labeling are as described for Additional file 12: Figure S12. (JPG 34 kb) [file 12906_2016_1423_MOESM14_ESM.jpg]

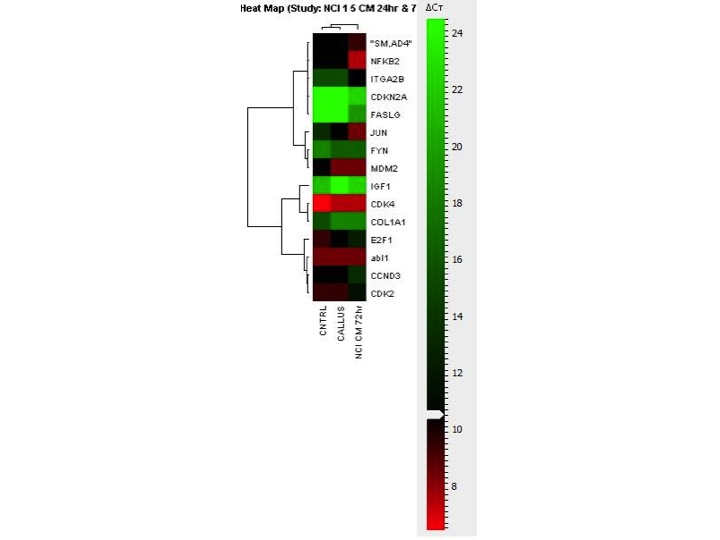

Supplement: Additional file 15: Figure S15. — Heat map representing expression levels of genes associated with cancer in NCI-H460 treated with 1:5 dilution RCSC for 72 h. The ΔCt was compared with untreated control. The red bar represents upregulation and green downregulation. Heat map was generated using Pearson correlation. Callus and NCI CM 72 h represent samples treated with RCSC 1:5 dilution for 24 h and 72 h, respectively. (JPG 35 kb) [file 12906_2016_1423_MOESM15_ESM.jpg]

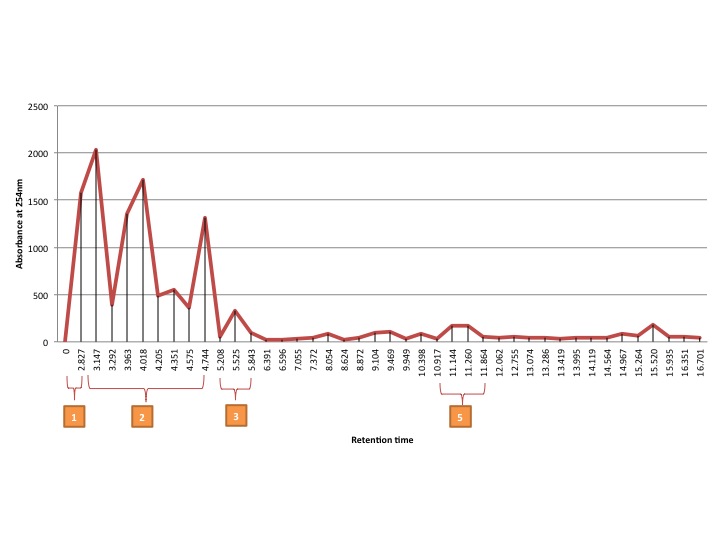

Supplement: Additional file 16: Figure S16. — HPLC chromatogram showing peaks at 254 nm. HPLC fractionation of RCSC showing peak heights on y-axis and retention time on x-axis. Fractions 1, 2, 3, and 5 selected for treatment of the lung cancer cell line are shown in the figure. (JPG 51 kb) [file 12906_2016_1423_MOESM16_ESM.jpg]

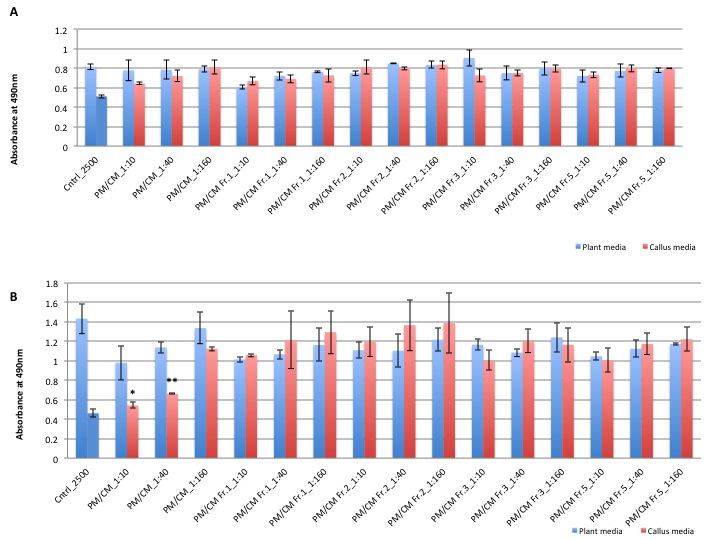

Supplement: Additional file 17: Figure S17. — Total LDH absorbance of NCI-H460 treated with RCSC and its HPLC fractions. Total LDH absorbance of NCI-H460 treated with five HPLC fractions of RCSC and plant media for (A) 24 h, (B) 48 h, (C) 72 h, and (D) 96 h. The x-axis shows different dilutions of plant media (PM), plant media fractions (PM fr.1, PM fr.2, PM fr. 3, PM fr.5), RCSC or callus media (CM) and callus media fractions (CM fr.1, CM fr.2, CM fr. 3, CM fr. 5). The y-axis shows average absorbance at 490 nm of LDH assay. Error bars represent standard deviation. Statistically significant effect of different dilutions of callus culture on the viability of cell lines is indicated by *p < 0.05 and **p < 0.01. Control represents untreated cell line and Med represents media without cells. (ZIP 185 kb) [file 12906_2016_1423_MOESM17_ESM.zip › Fig S17aR1.jpg]

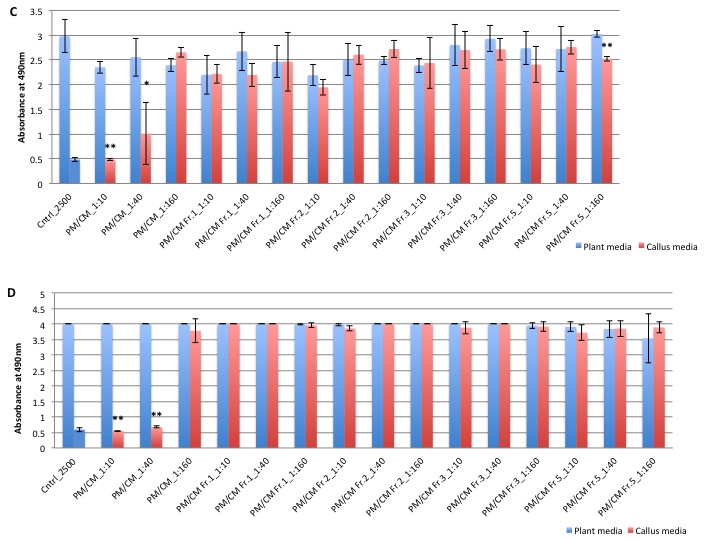

Supplement: Additional file 17: Figure S17. — Total LDH absorbance of NCI-H460 treated with RCSC and its HPLC fractions. Total LDH absorbance of NCI-H460 treated with five HPLC fractions of RCSC and plant media for (A) 24 h, (B) 48 h, (C) 72 h, and (D) 96 h. The x-axis shows different dilutions of plant media (PM), plant media fractions (PM fr.1, PM fr.2, PM fr. 3, PM fr.5), RCSC or callus media (CM) and callus media fractions (CM fr.1, CM fr.2, CM fr. 3, CM fr. 5). The y-axis shows average absorbance at 490 nm of LDH assay. Error bars represent standard deviation. Statistically significant effect of different dilutions of callus culture on the viability of cell lines is indicated by *p < 0.05 and **p < 0.01. Control represents untreated cell line and Med represents media without cells. (ZIP 185 kb) [file 12906_2016_1423_MOESM17_ESM.zip › Fig S17bR1.jpg]
